# Supplementary material for: Health effects of occupational noise exposure on heavy-duty equipment operators and exposed workers in a mining firm in Ghana
Source: PLoS One. 2025 Sep 19;20(9):e0332600. doi: 10.1371/journal.pone.0332600 (PMC12448357; doi:10.1371/journal.pone.0332600)
Supplement: S1 File — (DOCX) [file pone.0332600.s001.docx]

**QUESTIONNAIRE**

**A. SOCIO-DEMOGRAPHIC & WORK-RELATED FACTORS**

1. Age? (In years) …………………………………….
2. Gender Male { } Female { }
3. What is your religion? Christian { } Muslim { } Traditionalist { } Other
4. Educational level? No formal education { } Basic { } Secondary { } Tertiary { }
5. Marital status? a. Single { } b. Married { } c. others………………
6. Mining experience (years of work in the mining firm)? ……………………….. years
7. Work status? a. Employee { } b. Contract { } c. Casual { } d. other { } specify……………
8. Work department ……………………………..
9. How many hours per shift do you work?..............................................
10. How many days do you work in a week?.............................................
11. Have you had training on occupational health and safety since you started working here? Yes { } No { }
12. Do you follow the health and safety policies when working? Yes { } No { }
13. If yes, how many times within the year? …………………….
14. Do you have a rest room? Yes { } No { }
15. Does your work expose you to whole-body vibration? a. Never { } b. Occasionally { } c. <2hrs per day { } d.>2hrs per day
16. Does your work expose you to a lot of noise? a. Never { } b. Occasionally { } c. <2hrs per day { } d.>2hrs per day
17. Do you wear hearing protection devices during work? Yes { } No { }
18. If yes, how often do you wear it during work? a. Always { } Sometimes { }

**B. KNOWLEDGE ABOUT NOISE-INDUCED HEARING LOSS**

1. Are you aware of noise-induced hearing loss? Yes { } No { }
2. Where did you hear it from? a. Television { } b. radio { } c. workshops d. graphics e. other { } specify ……………..
3. Exposure to excessive noise can result in noise-induced hearing loss Yes { } No { }
4. Noise-induced hearing loss can be corrected medically and surgically Yes { } No { }
5. Noise-induced hearing loss can contribute to workplace injuries and accidents. Yes { } No { }
6. Which of the following can help to reduce noise-induced hearing loss at the workplace?
   1. Getting closer to the source of noise { }
   2. Prolonged exposure to loud noise { }
   3. Frequently taking breaks to limit the duration of exposure to loud noise { }
   4. Using the appropriate Personal Protective Equipment (PPE) eg; earplugs { }
   5. Developing of good equipment maintenance culture { }
7. Which of the following is the World Health Organization’s recommended noise exposure limit?
   1. 70 dB over a 24-hour period and 85 dB over a 1-hour period { }
   2. 60 dB over a 24-hour period and 85 dB over a 2-hour period { }
   3. 85 dB over a 24-hour period and 85 dB over a 3-hour period { }
8. A person with a noise-induced hearing loss can have a problem with hearing high-pitched sounds. Yes { } No { }
9. A person with noise-induced hearing loss will not have trouble understanding conversations when you are in a noisy place, such as a restaurant. Yes { } No { }
10. Degeneration of inner ear structures occurs over time and is associated with noise-induced hearing loss. Yes { } No { }
11. A symptom of noise-induced hearing loss does not include asking others to speak more slowly and clearly. Yes { } No { }
12. A person with a mild hearing loss may hear some speech sounds but soft sounds are hard to hear { } No { }

**C. HEALTH-RELATED PROBLEMS AND OCCUPATIONAL NOISE EXPOSURE**

1. Have you experienced any health-related problem in the last 12 months?
2. If yes in above, indicate by ticking any of the following health-related problems you have been diagnosed/experienced.

Sleepy difficulty { }

Hearing difficulty { }

Hearing loss { }

Ear infections { }

Ringing in the ears { }

Hypertension { }

Other { }, please specify ______________

1. Average exposure to noise in the last six months at the workplace__________________
2. Average percentage hearing loss reading in the past six months at the workplace_______
