# Supplementary material for: Health effects of occupational noise exposure on heavy-duty equipment operators and exposed workers in a mining firm in Ghana
Source: PLoS One. 2025 Sep 19;20(9):e0332600. doi: 10.1371/journal.pone.0332600 (PMC12448357; doi:10.1371/journal.pone.0332600)
Supplement: Attachment — Submitted filename: PONE-D-24-02308.docx [file pone.0332600.s003.docx]

PONE-D-24-02308

The authors have investigated the association between exposure to noise and experiencing work-related health outcomes. This study is relevant to the current state of the mining sector in Ghana, and I highly recommend it be published. The manuscript is well written however I recommend that the authors consider the following and refine the manuscript before publication.

1. There is an inconsistency noted within the manuscript concerning the participants of the study. While it is frequently indicated that the respondents were operators of heavy-duty machinery, Table 1 enumerates individuals from departments such as HSE, which do not engage in the operation of such equipment. The authors must clarify and reconcile this discrepancy.
2. I would suggest that the authors include the questionnaire used in their study as a supplementary file. This would enhance the transparency and reproducibility of the research.
3. A minor textual amendment is necessary on Page 5, Line 128, where the term "operators" should be inserted after "heavy-duty equipment" to maintain clarity in the description of the subjects.
